# Gilchrist’s Hollow Lung

**DOI:** 10.7759/cureus.44288

**Published:** 2023-08-28

**Authors:** Dedeepya Gullapalli, Avinash Vangara, Subramanya Shyam Ganti, Sai S Kommineni, Tara Rahmlow, Jessica Moon

**Affiliations:** 1 Internal Medicine, Appalachian Regional Healthcare (ARH), Harlan, USA; 2 Internal Medicine/Pulmonary Critical Care, Appalachian Regional Healthcare (ARH), Harlan, USA; 3 Internal Medicine, Lincoln Memorial University (LMU), Harrogate, USA

**Keywords:** methicillin-resistant staphylococcus aureus (mrsa), marijuana use, cavitary lung lesion, endemic disease, pulmonary blastomycosis

## Abstract

Blastomycosis is an endemic mycosis in certain parts of North America. The dimorphic fungus can manifest with both pulmonary and extrapulmonary features. We present the case of a 24-year-old African American male with a history of vaping and daily marijuana who presented with hemoptysis and a cough of one-week duration. He was initially treated as community-acquired pneumonia (CAP). The patient had a bronchoscopy with bronchoalveolar lavage (BAL) done in the posterior segment of the right upper lobe. Cultures grew methicillin-resistant *Staphylococcus aureus* (MRSA), followed by *Blastomyces dermatitidis* in the histopathologic examination. Chronic pulmonary blastomycosis may present with hemoptysis, weight loss, chronic cough, and night sweats, along with upper lobe predominant cavitation. We have to exclude tuberculosis (TB), lung cancer, and chronic pulmonary histoplasmosis. This case epitomizes many classic perils in the identification of pulmonary blastomycosis. The patient was being treated with itraconazole 200 mg BID for 12 months as per infectious disease suggestion. The patient is nine months into treatment. At six months, his chest computed tomography (CT) revealed a reduction in size from 5.0 × 5.3 cm to 4.2 × 4.0 cm. Although there are no articles supporting increased secondary bacterial infections with underlying fungal infections, more research needs to be done to find any associated features.

## Introduction

*Blastomyces dermatitidis* was first described by Thomas Caspar Gilchrist as a skin disease. It was initially identified as a protozoan but was later re-identified as a fungus. Hence, it is called Gilchrist disease [1]. It is a thermally dimorphic fungus that causes a systemic pyogranulomatous infection called blastomycosis. Unlike other deep fungal infections that occur primarily in immunocompromised patients, blastomycosis can also occur in immunocompetent hosts. It is endemic in the soils of the Ohio and Mississippi River Valleys, the Great Lakes region, and the southeastern United States. Most commonly, it presents as a pulmonary infection following the inhalation of spores. Infected patients may be asymptomatic or have life-threatening complications such as acute respiratory distress syndrome (ARDS), which mainly depends on the immune status of the patient.

## Case presentation

**Figure 1:**
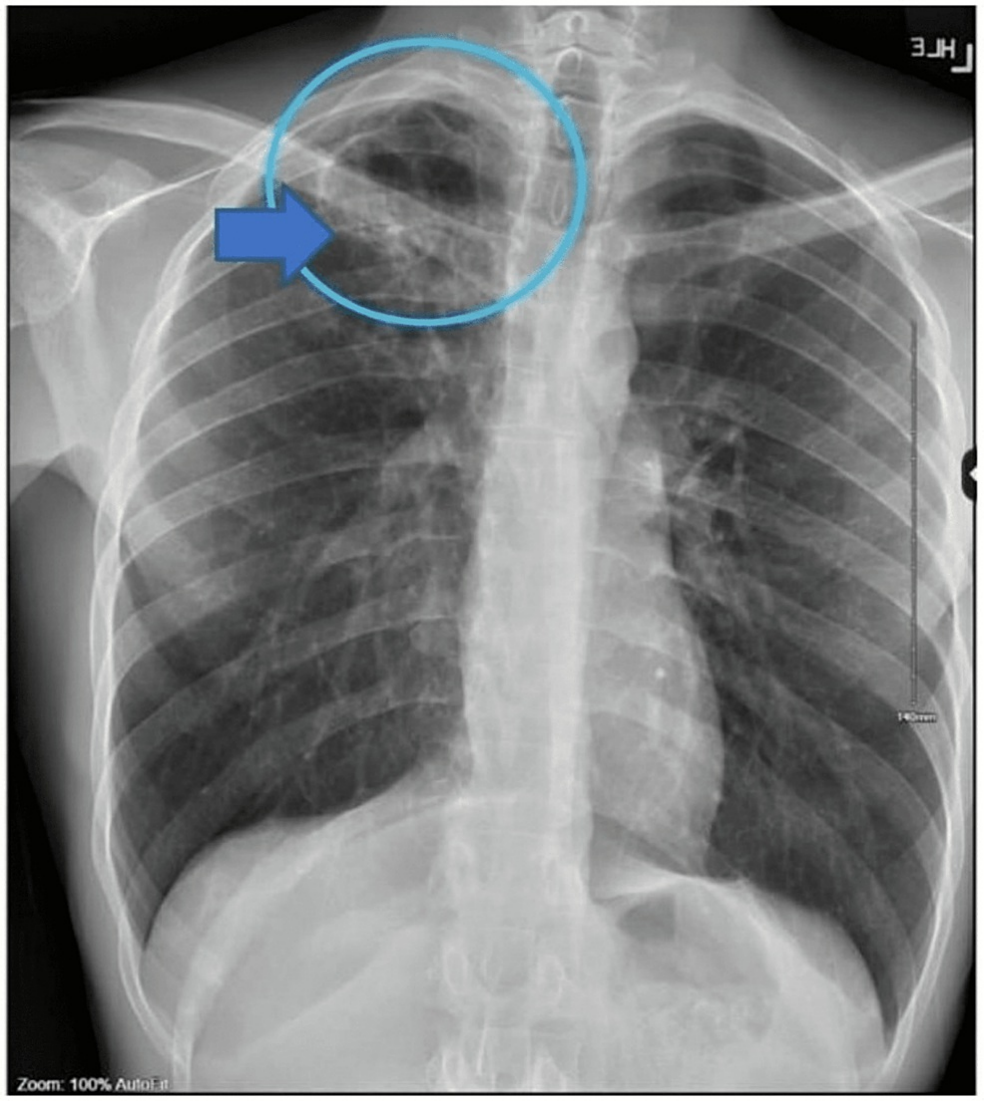
Chest X-ray showing findings of poorly defined infiltrates with a cavitary lesion in the right upper lobe (arrow)

A 24-year-old male with a past medical history of gastroesophageal reflux disease, asthma, marijuana usage, and vaping for the past six years presented to the emergency department with unrelenting cough and hemoptysis of one-week onset (day 0). He was hemodynamically stable. Physical examination revealed rhonchi and wheezing bilaterally. No skin lesions were noted. On the day of presentation, a chest radiograph (Figure 1) revealed right upper lobe infiltrates with findings suggestive of a cavitary lesion versus cyst versus bleb.

**Figure 2:**
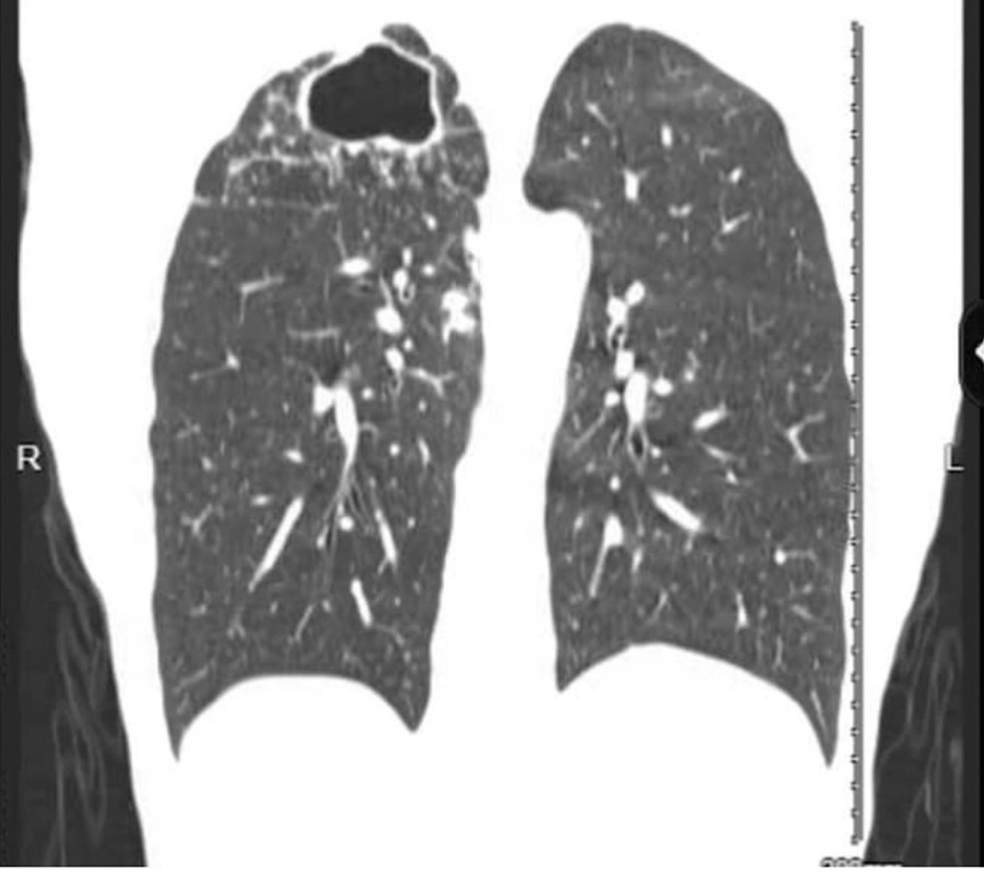
Chest CT revealing a 5.0 × 5.3 cm thick-walled cavitary lobe CT: computed tomography

**Figure 3:**
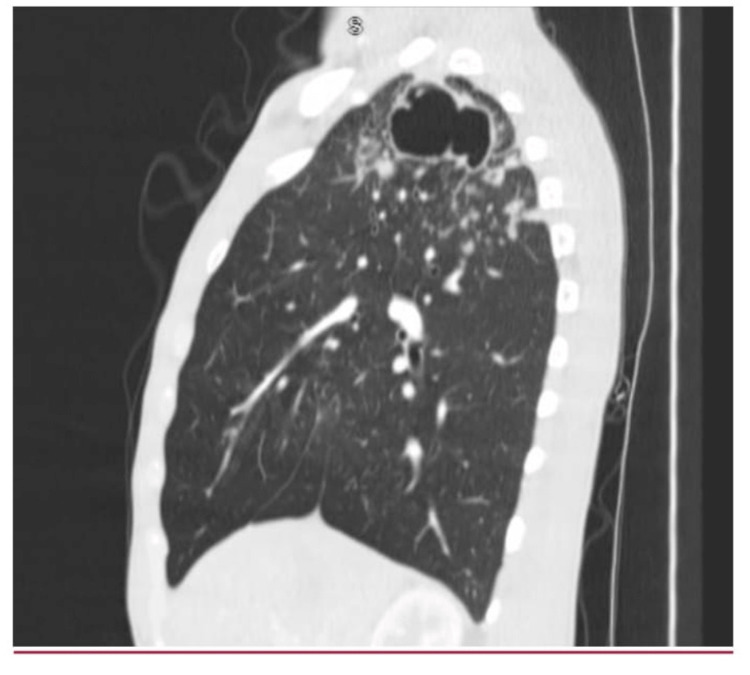
Chest CT revealing thick-walled cavitary lesion with satellite nodules adjacent to the cavitary mass CT: computed tomography

The patient was discharged from the emergency room (ER) with albuterol sulfate, Symbicort, and cefdinir from the ER with an outpatient pulmonology follow-up appointment. Two days later (day 2), the patient was evaluated by the pulmonologist for the cavitary lesion. On day 5, computed tomography (CT) scans of the chest (Figure 2 and Figure 3) demonstrated a large cavitary mass within the right lung apex with innumerable satellite nodular densities adjacent to the cavitary mass and, to a lesser extent, within the medial right lower lobe and left upper lobe.

**Figure 4:**
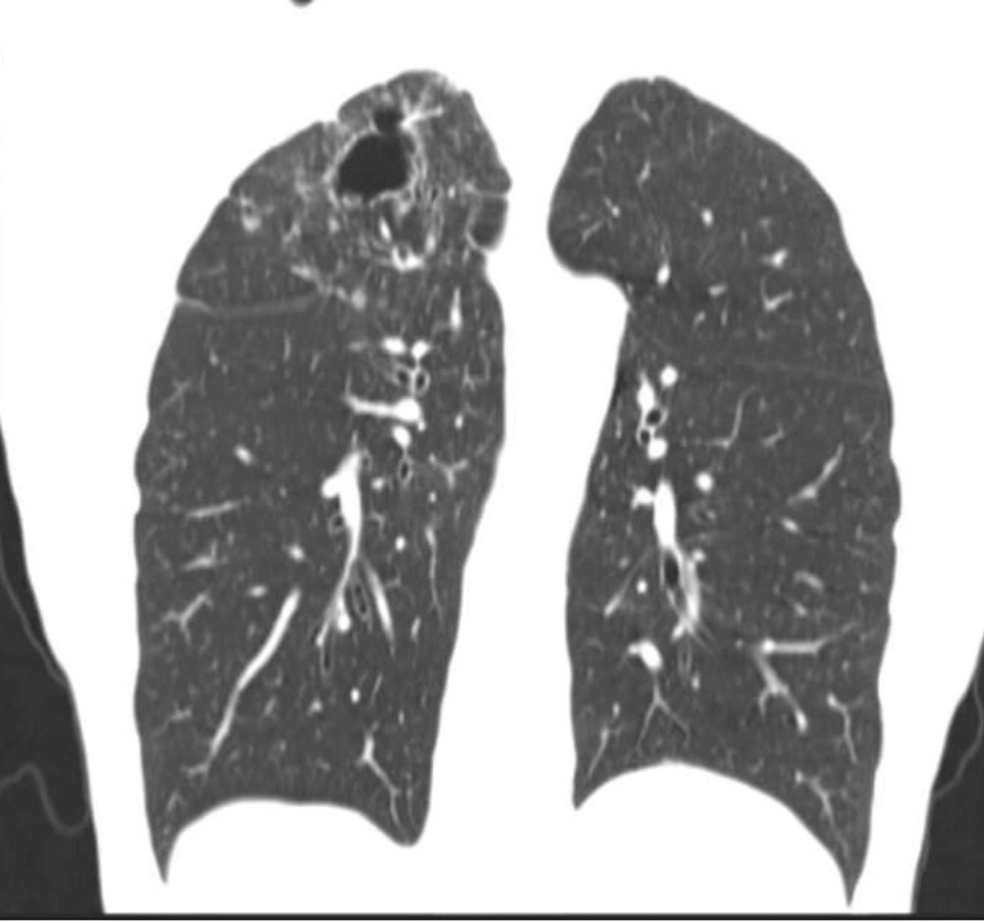
Repeat CT of the chest showing marked improvement in cavitary lesion with decreasing size and wall thickness in the right upper lobe CT: computed tomography

Differentials of tuberculosis (TB), fungal infections, and autoimmune conditions were considered. The patient denied a history of skin lesions, sick contacts, incarceration, and hiking, and he had a trip three months back to Massachusetts. On day 6, autoimmune workup including antinuclear antibodies and cyclic citrullinated peptide antibody, TB QuantiFERON, Fungitell, Aspergillus galactomannan antigen, Blastomyces antibody and antigen, Histoplasma antigen, and acid-fast bacillus sputum was ordered. All results were negative. On day 13, the patient mentioned that his cough was better and he had no hemoptysis. Two weeks later (day 20), a bronchoscopy with bronchoalveolar lavage was performed. During the procedure, blood-tinged mucus was noted in the posterior segment of the right upper lobe. Repeat serum Histoplasma and Blastomyces antigens were ordered as the patient is from an endemic zone for blastomycosis and histoplasmosis. Gram stain of the bronchial washings revealed numerous leukocytes. Three days later (day 13), the bronchial washings culture grew methicillin-resistant *Staphylococcus aureus*, and the patient was treated with one week of linezolid. One week later (day 27), serum Histoplasma antigen came back positive. Sixteen days later (day 36), bronchial washing cultures grew *Blastomyces dermatitidis* along with the serum Blastomyces antigen, which came back positive. Based on the diagnostics, symptoms, and course of the infection, the patient was diagnosed with pulmonary blastomycosis. He was started on itraconazole 200 mg BID on day 36 and referred to infectious disease with a plan for continuing treatment for one year of azole therapy. Itraconazole serum levels were not monitored. A cerebral CT (day 36) (done as per the infectious disease doctor’s suggestion) was also ordered to rule out any intracranial and bone lesions, which revealed no acute intracranial process. Repeat CT of the chest in the follow-up visit six months (day 30) later showed (Figure 4) a decrease in the size of wall thickness and right upper lobe cavitary lesion. At this time, the patient mentioned that his breathing was getting better and no hemoptysis was noted.

## Discussion

Blastomycosis is an endemic dimorphic fungus initially thought of as a protozoan by Thomas Caspar Gilchrist, most common in Ohio and Mississippi River basins. Its yearly incidence rate is 1-2 cases per 100,000 [2]. Pulmonary involvement is seen in 79% of patients infected with blastomycosis [3]. Lungs get involved mainly through inhalation of the conidia, which either disseminates or directly inoculates the cutaneous tissue. It ranges from subclinical pneumonia to acute respiratory distress syndrome (ARDS) depending on the immune status of the patient [3]. Consolidation being the most common radiological feature, acute pulmonary blastomycosis can be confused with community-acquired pneumonia (CAP) [4]. Chest radiograph studies showed the prevalence of consolidation being 26%-51% [5].

Our patient was initially diagnosed with CAP and treated with cefdinir from the ER, and once the BAL cultures came back positive for MRSA, he was treated with linezolid. Untreated or undiagnosed acute pulmonary blastomycosis may progress to chronic pulmonary blastomycosis or ARDS. Symptoms include fever, chronic cough, hemoptysis, weight loss, and decreased appetite [3]. Our patient presented with a chronic cough associated with hemoptysis and shortness of air. Rupture of the pulmonary or bronchial vasculature is the cause of hemoptysis. As blastomycosis has a high mortality rate, especially in endemic areas, a suspicion for this disease as a differential should be kept in mind [6]. Chest radiography shows nodules, mass, or cavity. Major pulmonary radiological features include consolidation, intermediate-sized nodules, cavitation (62% roughly), lymph node enlargement (32%), and cavitary lesions (29%) [5,7]. Our patient had a right upper lobe patchy infiltrate and cavity. Diagnosis is usually delayed because of the overlap of symptoms with other common pulmonary diseases. In a case like this, laboratory, imaging, and bronchoalveolar lavage culture findings lead to the diagnosis of blastomycosis. A study from the Journal of Clinical Microbiology found the sensitivity of these assays in pulmonary blastomycosis to only be 64.3% in the serum, 82.7% in urine, and 62.5% from BAL sampling [8]. The definitive diagnosis is made by visualization of the organism in histology-positive culture findings from the specimen. Under gram stain or periodic-acid Schiff (PAS) stain, it is seen as single broad-based budding yeast cells [9]. More specific nucleic acid detection, polymerase chain reaction, and repetitive DNA are useful in more complicated cases [3].

Treatment should be initiated as early as the patient is diagnosed with blastomycosis and should not be delayed. Mild to moderate blastomycosis is treated with oral itraconazole for 6-12 months [3]. Differential diagnosis includes other systemic fungal infections, tuberculosis (TB), malignancy, and lung abscesses. Other fungal infections including histoplasmosis can be differentiated with extrapulmonary manifestations; antigen testing for disseminated histoplasmosis is more sensitive in urine (95%) compared to serum (86%), and a combination of antigen-antibody testing increases the sensitivity to 96% [10]. However, there is high cross-reactivity between blastomycosis and histoplasmosis antigen assays, and 60% of urine blastomycosis antigens cross-reacting with histoplasma antigen assay and cross-reactivity between antibodies is also noted [11]. Cytology and cultures are diagnostic. Based on the literature on fungal infections and cannabis use, the proposed hypothesis was of immunocompromised status and hospitalizations. People who used cannabis were comparatively sicker than those who did not use cannabis and were therefore presumably at higher risk for fungal infections in general [12].

## Conclusions

In conclusion, blastomycosis can occasionally present with clinical and radiographic features indistinguishable from thoracic malignancies and community-acquired pneumonia. Definitive diagnosis is achieved by visualizing the organism by histopathologic examination or obtaining a positive culture. We have to consider the differential of blastomycosis especially in endemic areas. Early diagnosis and treatment can prevent the complications of ARDS in patients. Although there are no articles supporting increased secondary bacterial infections with underlying fungal infections, more research needs to be done to find any associated features. More literature should be proposed about blastomycosis in marijuana users.
